# Evaluation of Shear Bond Strength and Failure Modes of Lithium Disilicate Ceramic Veneering Material to Different High-Performance Polymers

**DOI:** 10.3390/polym17050554

**Published:** 2025-02-20

**Authors:** Sarah M. Alnafaiy, Nawaf Labban, Refal Albaijan, Rawan N. AlKahtani, Khulud A. Al-Aali, Heba Wageh Abozaed, Nada Y. Alturki, Jomana E. Alenezi

**Affiliations:** 1 Department of Clinical Dental Sciences, College of Dentistry, Princess Nourah bint Abdulrahman University, Riyadh 11671, Saudi Arabia; rnalkahtani@pnu.edu.sa (R.N.A.); kaalaali@pnu.edu.sa (K.A.A.-A.); 2 Department of Prosthetic Dental Sciences, College of Dentistry, King Saud University, Riyadh 11545, Saudi Arabia; nalabban@ksu.edu.sa; 3 Department of Prosthodontics, College of Dentistry, Prince Sattam bin Abdulaziz University, Al-Kharj 11942, Saudi Arabia; r.albaijan@psau.edu.sa (R.A.); heba_zeid@mans.edu.eg (H.W.A.); 4 Department of Prosthodontics, Faculty of Dentistry, Mansoura University, Mansoura 35516, Egypt; 5 Dental Intern, College of Dentistry, Princess Nourah bint Abdulrahman University, Riyadh 11671, Saudi Arabia; 439002444@pnu.edu.sa (N.Y.A.); 439001744@pnu.edu.sa (J.E.A.)

**Keywords:** PEEK, BioHPP, high-performance polymers, glass fibers, SBS, ceramic veneer

## Abstract

This study assessed the shear bond strength (SBS) and failure modes of lithium disilicate ceramic veneering material to different high-performance polymers. Thirty-six square specimens measuring 7 × 7 × 2 ± 0.05 mm were prepared from pure polyetheretherketone (PEEK), Bio-high performance PEEK (BioHPP) and Trilor discs. Polymer specimens were air-borne abraded utilizing aluminum oxide particles, cleaned, and a bonding agent was applied (visio. link). The veneering LDC material (3 × 2 mm) was milled, hydrofluoric acid etched (9.5%) and primed (Clearfil ceramic). The LDC was bonded to the polymer specimens using dual-cured resin cement (Panavia V5) and light polymerized. The bonded specimens were subjected to 5000 cycles of physiological aging by thermocycling, and the SBS test was performed in a universal testing machine at 0.5 mm/min cross-head speed. The debonded specimens were analyzed to determine the primary bond failure sites (adhesive, mixed or cohesive). Data analysis was performed using one-way ANOVA and a post hoc Tukey test (α ≤ 0.05). The BioHPP material demonstrated the highest SBS values (23.94 ± 1.43 MPa), and the Trilor group recorded the lowest SBS values (17.09 ± 1.07 MPa). The PEEK group showed a mean SBS of 21.21 ± 1.51 MPa. The SBS comparison showed significant variations across all material groups (*p* < 0.001). Regarding failure modes, adhesive failure was observed in 40% of BioHPP and PEEK specimens and 90% of Trilor specimens. The cohesive failure occurred in 50% of PEEK and 30% of BioHPP specimens, while the Trilor specimens showed no cohesive failure. Mixed failures were reported in 30% of BioHPP and 10% of PEEK and Trilor specimens. The BioHPP material demonstrated high SBS followed by PEEK and Trilor. The SBS between the tested materials was statistically significant. However, the SBS of the tested implant framework materials was above the limit stipulated by the ISO 10477 standard (5 MPa) and the clinically acceptable range of 10–12 MPa.

## 1. Introduction

Rigid metal materials such as titanium and cobalt–chromium were commonly used to fabricate implant frameworks due to their excellent mechanical and biocompatibility properties [1,2]. However, some esthetic complications were reported, including the possibility of degradation, corrosion, and casting distortion that could reduce the accuracy and fit of the framework [2,3,4]. In different studies, prosthetic maintenance complications were also reported, including framework fracture, screw loosening or fracture, veneered material chipping, wear, and fracture of resin denture teeth [5,6,7].

A metal–ceramic hybrid prosthesis was then developed to overcome some of the complications of the acrylic–metal prosthesis [8,9]; however, ceramic chipping was one of the most reported technical complications with this hybrid combination [9]. Furthermore, ceramic reparability is considered more time-consuming and complicated than acrylic, and indirect intervention through a laboratory may be required [10]. Another disadvantage is related to the ceramic firing, which affects the accuracy and fit of the framework to the implants [11,12].

The introduction of computer-aided design and computer-aided manufacturing (CAD-CAM) technology in dentistry has replaced the conventional workflows, including framework casting, and has also reduced casting errors and complications [8,9]. This technology is reported to decrease the visits for clinical and laboratory procedures while providing better mechanical and physical properties than those obtained by the conventional technique. Additionally, the method can be applied to materials like zirconia, cobalt–chromium alloys, and titanium and its alloys [13]. Furthermore, the data and records of the prosthesis are saved and they can be accessed readily if a new prosthesis is to be fabricated in case of a fractured or lost prosthesis, thereby reducing overall patient treatment costs [14,15]. CAD-CAM technology has facilitated using non-castable materials such as zirconia as a full-arch implant framework because of its favorable biological and mechanical properties [16,17]. Zirconia combines the aesthetics of glass-matrix ceramics and the strength of metals [18]. However, zirconia is a rigid material with a modulus of elasticity of ~210 GPa, which is significantly higher than the modulus of elasticity of cortical bone (~13 GPa) [19]. This discrepancy in elasticity could increase the overall local stress concentration between the zirconia implant and peri-implant bone, leading to peri-implant bone loss [19,20].

Polyetheretherketone (PEEK) as a material has been developed for hybrid implant prosthesis [21,22]. PEEK is a semi-crystalline, thermoplastic material with a high melting temperature, which is indicated mainly to be used as an implant framework [23]. The elastic modulus of PEEK is 3.6 GPa and can reach up to 12–18 GPa if reinforced by materials such as carbon or glass fibers [24]. The reinforced PEEK’s elasticity is considered close to cortical bone, acting as a shock absorbent for an implant framework material [25,26]. A PEEK prosthesis has low weight, providing the patient with more comfort and causing less wear on the opposing teeth [27,28]. However, the disadvantage of this material is that it is not bioactive, and its strength is low compared to metal and zirconia materials [29]. Moreover, its poor translucency and white-grayish color hinder it from being used in dental restorations, particularly for aesthetic reasons [30]. Therefore, it needs to be veneered with ceramic-based materials to achieve an acceptable aesthetic outcome [30,31].

A PEEK variant, namely Bio-high performance PEEK (BioHPP), has 20% ceramic particles embedded in PEEK and has demonstrated remarkable physical properties compared to other implant framework materials [28]. The material’s physiologic integration, resistance to plaque, light weight, elasticity (about 4 GPa), flexural strength of >185 MPa and fracture strength of 700–1600 N are all perfectly balanced, and its flawless surface allows for polishability to <0.02 µm [32,33]. BioHPP has been used as a full-arch implant framework material with composite veneer and demonstrated a significantly high bond strength compared to a titanium framework [34]. It also exhibited good marginal fit and fracture resistance that was comparable to a metal framework [28,32].

Recently, another high-performance, tooth-colored and biocompatible polymer (Trilor, Bioloren S.r.l, Saronno VA, Italy) has been introduced. This epoxy material is reinforced with multidirectional glass fibers and is mainly suggested for the CAD-CAM fabrication of removable partial dentures due to its better flexural properties than PEEK (540 MPa vs. 150 MPa) [35,36]. It is also recommended for the milling of frameworks for aesthetic tooth and implant-supported restorations veneered with composites, ceramics and zirconia because of its bone-like durability [37].

The quality of adhesion is evaluated using bond strength testing. A satisfactory functional outcome and long-term stability between the veneering and framework materials depend on understanding the bonding characteristics [38]. Different factors have been reported to affect the bond strength between the veneering and framework materials, such as the adhesive systems, surface preparation and material composition [39]. In different studies, polymer-based framework materials veneered with composite resin showed higher bond strengths compared to metal or zirconia materials due to their high bonding affinity with the silicate particles of the cement and composite resin [30,40,41]. However, studies evaluating and comparing the shear bond strength (SBS) of lithium disilicate ceramic (LDC) to different polymer-based framework materials are scarce, especially with the recently introduced BioHPP and Trilor materials. One study by Sloan et al. [42] has evaluated the bond between PEEK and LDC. To the best of the author’s knowledge, there is no literature regarding the bond strength of LDC to BioHPP or Trilor materials at the time of this study.

Thus, this study aims to assess and compare the SBS of the LDC veneering material to different high-performance polymers. The null hypothesis is that there is no significant difference in the SBS of the three polymer-based implant framework materials veneered with the LDC material.

## 2. Materials and Methods

The SBS of the lithium disilicate ceramic veneering material to three polymer-based implant framework materials—PEEK, BioHPP and Trilor—was assessed, and the predominant failure site was categorized in the present study. The material details are listed in Table 1. The Institutional Review Board at the Princess Nourah bint Abdulrahman University (HAP-01-R-059) approved the research protocol. Table 2 presents the flexural strength and elastic modulus of the materials tested [43,44].

### 2.1. Sample Preparation

The sample size for this study was from previous studies evaluating the SBS of veneering materials to polymer-based materials [30,45]. Thirty-six square specimens measuring 7 × 7 × 2 ± 0.05 mm were sectioned from PEEK, BioHPP and Trilor (n = 12) polymer discs using an automated saw (IsoMet, Buehler, IL, USA) under water coolant [19]. For the veneering material, 3 × 2 mm LDC discs were milled using an IPS E.max CAD blank in a CAD/CAM milling apparatus (Ceramill Motion 2, Amann Girrbach AC, Koblach, Austria). The dimensions of the prepared polymer specimens and LDC veneering materials were confirmed by a digital micrometer (Digimatic Micrometre, Mitutoyo, Japan). The polymer specimens were individually ingrained into locally available rigid PVC tubes (Ø 10 mm) using self-polymerizing clear acrylic resin (Takilon, Rodent, Milano, Italy). For standardization of the bonding surface of all the specimens, the surfaces were polished (LaboPol, Struers, Copenhagen, Denmark) at 300 rpm for 30 s under water-coolant, utilizing 500-grit waterproof silicon carbide papers (Struers, Copenhagen, Denmark). The polished specimens were cleaned ultrasonically (Quantrex^®^, L&R Manufacturing, Inc., Kearny, NJ, USA) for 15 min and then air dried for 30 s.

### 2.2. Surface Treatment

Following the manufacturer’s recommendations, the polymer-based specimen’s bonding surface was air-borne abraded utilizing aluminum oxide particles (110 μm) from a distance of 10 mm, at 2 bar PSI for 15 s (Basic; Renfert, Hilzingen, Germany). The surface-treated specimens were cleaned ultrasonically, containing deionized water for 5 min. The bond surface of the LDC was 9.5% hydrofluoric acid etched for 20 s (Bisco Inc., Schaumburg, IL, USA), water-rinsed and air-dried. A single primer layer was applied to the etched surface (Clearfil Ceramic Primer Plus, Kuraray America, Inc., New York, NY, USA) and allowed to dry.

### 2.3. Bonding Procedure and Aging

The predetermined bonding surface of the polymer specimens was coated with a single, thin layer of bonding agent (visio. link; Bredent Medical GmbH & Co KG, Senden, Germany) according to the manufacturer’s recommendation. The bonding agent was light activated using a hand-held device (Elipar Free Light, 3M ESPE, Bayern, Germany) at a 420–540 nm wavelength for 90 s. The LDC veneering material was cemented onto the polymer specimen’s surface using dual-cured resin cement (Panavia V5; Kuraray Europe GmbH, Hattersheim am Main, Germany) confined via a translucent polyethylene matrix. Light finger pressure was applied on the LDC material that caused extrusion of the excess resin cement, which was carefully removed by an explorer. The specimen was light-polymerized for 30 s.

**Figure 1:**
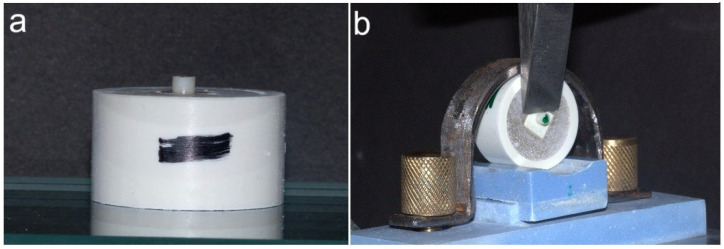
(**a**) LDC veneering material bonded to polymer specimen, (**b**) SBS test set-up.

The bonded specimens (Figure 1a) were immersed in distilled water at room temperature for 24 h and then artificially aged by thermocycling (SD Mechatronik GmbH, Feldkirchen-Westerham, Germany) for 5000 cycles. The thermocycling parameters were as follows: 5–55 °C water temperature bath, 30 s dwell time and transfer time of 10 s between the water bath [46]. The thermocycling parameters were in accordance with DIN EN ISO 10477 guidelines [47]. The ISO guidelines recommend a minimum of 500 cycles, but in the current study, 5000 cycles were applied to simulate six months of clinical use [48].

### 2.4. Shear Bond Strength (SBS) Test

The specimens were placed and secured utilizing a customized holder in a universal testing apparatus (Model 5965, Instron^®^ Corporation, Norwood, MA, USA) (Figure 1b). The shearing rod was advanced at a constant speed of 0.5 mm/min, directed towards the long axis of the adhesion surface until fracture. As the loading rod and speed were controlled by the software connected to the universal testing apparatus, the effect of external factors affecting the shearing rod was minimal. The resulting force that caused failure was obtained in Newtons (N) and then calculated to megapascals (MPa) by using the Formula below (1) [30]:

SBS (MPa) = Debonding force (N)/adhesive surface area (mm^2^) (1)

### 2.5. Evaluation of Failure Modes

After the SBS test, the failure sites were examined to assess the failure modes using a digital microscope (Hirox-USA Inc., Oradell, NJ, USA) under ×20 magnification and a 1000 µm surface area. The primary failure modes were categorized as either adhesive (failure occurring between the polymer and LDS), cohesive (failure occurring within the LDS or polymer materials) or mixed (combination of adhesive and cohesive failure) [40].

### 2.6. Statistical Analysis

Data were analyzed using SPSS (v.22, IBM SPSS Inc., Chicago, IL, USA). The Shapiro–Wilk test verified the normality of data (α = 0.05). One-way ANOVA was used to compare the difference in the SBS between the three groups followed by a post-hoc Tukey test, which was applied to determine the significant difference in the SBS between the groups (α = 0.05).

## 3. Results

### 3.1. SBS Test

**Figure 2:**
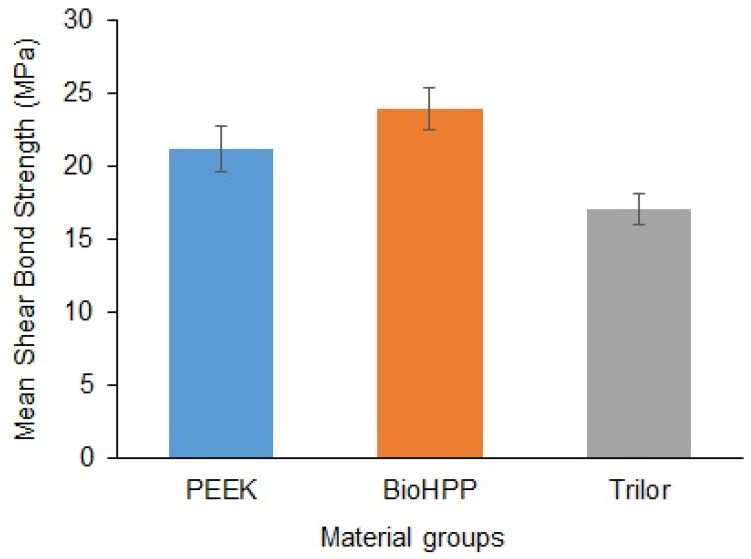
Mean SBS values of the material groups. Bars indicate SD.

The mean and SD values of the SBS (in MPa) of LDS veneer to different high-performance polymers are presented in Figure 2. The BioHPP group demonstrated the highest SBS values, with a mean of 23.94 ± 1.43 MPa. In contrast, the Trilor group recorded the lowest SBS values, with a mean of 17.09 ± 1.07 MPa. The PEEK group showed a mean SBS value of 21.21 ± 1.51 MPa. The SBS comparison revealed significant differences among all material groups (*p* < 0.001) (Table 3).

### 3.2. Failure Mode Analysis

**Figure 3:**
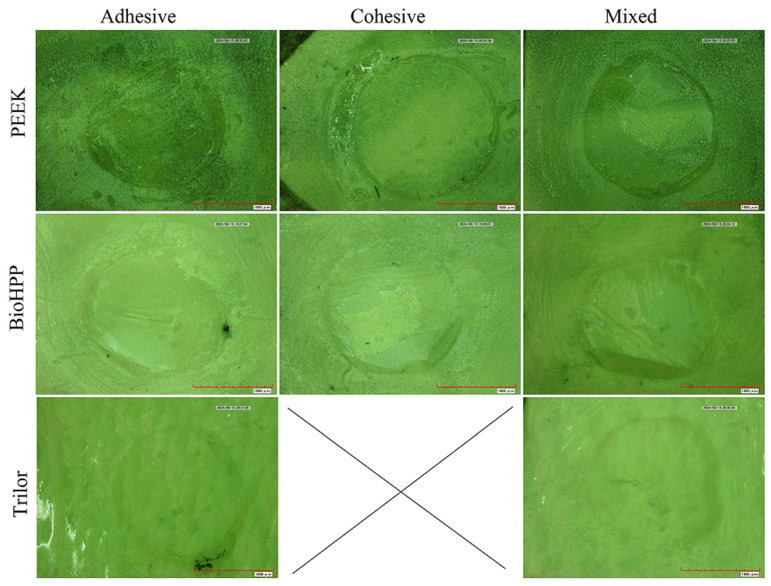
Microscopy image of representative specimen failure from each group.

All specimens were assessed for failure modes following the SBS measurements for each materials group. All types of failure modes—adhesive, cohesive and mixed—are summarized in Table 4. Figure 3 displays the microscopic image of representative specimen failure from each group. Most specimens demonstrated adhesive failure, followed by cohesive and mixed failures. Trilor specimens showed adhesive failures for 90% of the specimens, which substantially varied from the other materials.

## 4. Discussion

Millable CAD-CAM materials that have been recently introduced to the market for fabrication of implant-supported frameworks have gained significant interest among researchers and clinicians. In that context, the biomimetic characteristics and high strength of biocompatible high-performance polymers make them beneficial in the prosthetic replacement of oral hard tissues [37]. PEEK’s bond strength to composite resin and LDC’s bond to titanium and zirconia have been studied enormously. However, there is a dearth of research on the bond between LDC and polymer-based PEEK, and in particular, the recently introduced BioHPP and Trilor materials. The clinical use of these materials seems to have surpassed the literature in recent years. When addressing the problem of veneer material retention, it is critical to comprehend the characteristics of this bond [42]. The current in vitro study assessed and compared the SBS of an LDC veneering material to different high-performance polymers. It was hypothesized that there would be no significant difference in the SBS of the LDC veneering material to three polymer-based implant framework materials. The analysis of the SBS data showed a significant difference in the SBS between LDC and the tested polymer-based materials, thus recommending the rejection of the null hypothesis. However, the SBS obtained with all the three tested materials exceeded the optimal SBS of 5 Mpa, as stipulated by the ISO 10477 standard and the clinically acceptable range of 10–12 MPa. This suggests that all the three materials could be used to fabricate implant frameworks and to adequately bond to LDC in clinical scenarios.

Ceramic materials have successfully addressed the shortcomings of composite resin materials in terms of durability, color and wear resistance. In particular, LDC has shown impressive outcomes and has been successfully used as a prosthetic material in dentistry. It offers exceptional mechanical and optical qualities. The LDC material has demonstrated adequate bonding with resin-based materials following surface treatment with hydrofluoric acid etching and silane application. The material, therefore, is recommended as a substitute for traditional PEEK veneering [49].

BioHPP is a 0.3–0.5-µm-sized ceramic particle reinforced PEEK with a homogeneous structure, which is reported to offer advanced mechanical properties [45]. The SBS data from this study showed that BioHPP had a comparatively and significantly high SBS of all the study groups. The material with the lowest SBS was Trilor, a techno-polymer reinforced with 75 wt% multidirectional glass fiber. Trilor’s ability to bend and flex under stress makes it an ideal milling composite for implant-supported restorations. The material has an elastic modulus of 26 GPa, which is close to the elastic modulus of bone (16–20 GPa) [50,51]. Therefore, it can be used to fabricate durable substructures for composites, ceramics and acrylic restoration [52]. According to the manufacturers of Trilor, the multi-directional braided fiber structure offers good performance in terms of load and tension distribution in response to forces applied from different angles, making it an ideal material for permanent and temporary dental restorations [43].

The polymer-based materials in this study demonstrated SBS values of 17.09–23.94 MPa, which exceeded the ISO 10477 standard’s recommended limit of 5 MPa. Additionally, the SBS values exceeded the clinically acceptable range of 10–12 MPa [45,49]. Kilic et al. [40] demonstrated that the SBS of LDC to PEEK was 13.88 ± 1.77 MPa, which was significantly low compared to the values reported in the current study. In another study by Gökay et al. [45], the SBSs of PEEK and BioHPP framework materials to Vitablocs Mark II, Cerasmart and Vita Enamic veneering materials were compared. The SBS of feldspathic ceramic Vitablocs Mark II to PEEK (4.82 MPa) was significantly low compared to BioHPP (7.98 MPa). However, a SBS interpretation between studies should be considered carefully. The filler concentration, crystallinity, free surface energy, surface roughness, contact angle, material chemistry and the surface treatment applied all affect the bond strength, a multifactorial characteristic [53,54].

The composition of the adhesive and the surface treatment are crucial for a durable bond. Bonding to PEEK and its variants is thought to be almost entirely micromechanical because of the unreactive nature of the PEEK surface [55]. This study confirms that the manufacturer recommendation of air-particle abrasion followed by primer application will result in adequate bond strength. In a previous study, Ruse et al. demonstrated that air-particle abrasion (50 µm aluminum oxide) of PEEK surfaces produced the highest bond strength compared to argon–oxygen plasma and non-thermal air plasma. On the contrary, Kilic et al. [40] demonstrated that a sulfuric acid treatment of PEEK surfaces produced the highest SBS to LDC compared to air-particle abrasion; although, the SBS was not significant between the groups. The impact of airborne particles enhances surface roughness and breaks the C-C and C-H polymer chains, enhancing the micro-mechanical bonding area as well as the bonding agent’s wettability and penetration within the polymer. Furthermore, the free radicals that are generated upon the breakage of the polymer chain may also strengthen the chemical bond with resin-based adhesives by initiating a chain transfer reaction with the adhesive agent [56].

Since BioHPP was developed to enhance the mechanical properties of PEEK, it is expected that it would also yield a higher SBS [45]. Accordingly, the reinforcement of PEEK by ceramic particles, as in BioHPP, could be possibly related to the high SBS compared to conventional PEEK. On the contrary, the exact reason for the low bond strength of Trilor compared to other materials remains unclear. One possible reason could be the sawing of the fiber-reinforced CAD specimens. The CAD blocks in most studies are sawed for practical purposes. This does not seem to be an issue with homogeneous materials like PEEK, whereas sawing is not suitable for fabricating specimens with multiphase, millable fiber-reinforced materials. Depending on the specimen, the multidirectional fiber networks may be sliced in different directions [57], which could affect the mechanical properties, including SBS.

One crucial factor in comprehending the results of SBS tests is the failure mode analysis [30]. The current study findings demonstrated that adhesive failures accounted for 57% of all failures, which constituted 90% of the Trilor specimens. This suggests that adhesive failures accounted for the majority of the complex polymer structures—resin cement–LDC—leaving the LDC discs free of adhesive material residues. This was followed by 26% of the specimens showing cohesive failures and 17% of the specimens showing mixed failures. It has been reported that there is a direct and positive correlation between bond strength and the percentage of cohesive failure [30]. This was true in the case of the Trilor specimens, with low SBSs and no cohesive failures compared to other groups.

Since routine oral processes induce thermal changes in the oral environment, thermocycling has been shown to be an appropriate method for simulating these changes [49]. Furthermore, SBS testing after thermocycling is the most common method of assessing the SBS of veneering materials to framework or substructure materials [30]. The specimens in this investigation were aged for 5000 cycles, which corresponds to six months of intraoral use, despite the fact that there is no exact method for thermocycling [48]. All examined samples were thus exposed to consistent and repeatable thermal stress [49]. It has already been demonstrated in previous studies that thermocycling has a significant effect on the adhesive bond between different materials [57]. Nevertheless, in the current study, these effect could not be extrapolated, as the SBS was measured only after thermocycling.

The outcome of this study is not without limitations. Despite the fact that this study was unable to reproduce all the intraoral conditions, it could be useful in establishing reliable bonding between veneering materials and polymer-based implant framework materials in dentistry. Routine oral processes such as brushing, chewing, and diet could have a significant and different effect on SBS, which were not considered in this study. The findings of this study could not be compared with other prior studies as this is the first study to evaluate the SBS of LDC to high-performance polymer materials. This necessitates further studies both in vitro and in vivo to clearly understand the properties of these new materials. It is important to evaluate the effect of routine oral habits (brushing and chewing) on the SBS of LDC bonded to high-performance polymers. Furthermore, the materials need to be assessed and compared in terms of other properties, including color stability, surface roughness, wettability and mechanical and physical properties, mainly in a dynamic oral environment.

## 5. Conclusions

Based on this study’s limitations, the following conclusions are drawn:

- This study demonstrated that the BioHPP material had a higher SBS, followed by the PEEK and Trilor materials, and the SBS between the materials was significantly different (*p* < 0.05).
- The SBSs of all the tested implant framework materials were above the limit stipulated by the ISO 10477 standard (5 MPa) and the clinically acceptable range of 10–12 MPa.
- All the tested materials could be used to fabricate implant frameworks and to adequately bond to LDC.
- The primary failure mode was adhesive, followed by cohesive and mixed failures.

## Figures and Tables

**Table 1:** Study materials.

Materials	Shade	Composition	Manufacturer
Copra Peek/Polyetherether ketone (PEEK)	Dentin	100% polyetheretherketone	Whitepeaks Dental Solutions GmbH, Weikenrott, Hamminkeln, Germany
BioHPP^®^/Ceramic reinforced high-performance polymer	Dentin	PEEK filled with 0.3–0.5 µm ceramic filler (20%)	Bredent GmbH & Co Kg, Senden, Germany
Trilor^®^/Thermoset resin reinforced with multi-directional fiber glass	Bone	25% epoxy resin matrix reinforced with 75% multi-directional glass fiber	Metal free dental system|Bioloren, Saronno VA, Italy
IPS E.max CAD/Low-translucent lithium disilicate (LLD)	A1	58–80% SiO_2_, 0–13% K_2_O, 11–19% Li_2_O, 0–8% ZrO_2_, 0–5% Al_2_O_3_, 0–8% ZnO, 0–5% MgO, 0–8% and coloring oxides	Ivoclar Vivadent AG, Schaan, Liechtenstein

**Table 2:** Flexural strength and elastic modulus of the materials tested.

Properties	PEEK	BioHPP	Trilor
Flexural strength	150 MPa	>150 MPa	540 MPa
Elasticity	4 GPa	40 GPa	26 Pa

**Table 3:** Comparison of SBS (in MPa) between the material groups.

Group (I)	Group (J)	Mean Difference (I-J)	Std. Error	*p*
PEEK	BioHPP	−2.72 *	0.60	<0.01 *
	Trilor	4.12 *	0.60	<0.01 *
BioHPP	PEEK	2.72 *	0.60	<0.01 *
	Trilor	6.84 *	0.60	<0.01 *
Trilor	BioHPP	−6.84 *	0.60	<0.01 *
	PEEK	−4.12 *	0.60	<0.01 *

* Statistically significant (*p* < 0.05).

**Table 4:** Failure modes of the groups.

Materials	Adhesive	Cohesive	Mixed (Both Adhesive and Cohesive)
PEEK	40%	50%	10%
BioHPP	40%	30%	30%
Trilor	90%	0%	10%

## Data Availability

All the data are present in the manuscript.

## References

[1] Hossain N., Mobarak M.H., Islam M.A., Hossain A., Al Mahmud M.Z., Rayhan M.T., Chowdhury M.A. (2023). Recent development of dental implant materials, synthesis process, and failure—A review. Results Chem..

[2] Přikrylová J., Procházková J., Podzimek Š. (2019). Side Effects of Dental Metal Implants: Impact on Human Health (Metal as a Risk Factor of Implantologic Treatment). BioMed. Res. Int..

[3] Bidra A.S., Rungruanganunt P., Gauthier M. (2017). Clinical outcomes of full arch fixed implant-supported zirconia prostheses: A systematic review. Eur. J. Oral Implantol..

[4] Saha S., Roy S. (2023). Metallic Dental Implants Wear Mechanisms, Materials, and Manufacturing Processes: A Literature Review. Materials.

[5] Cinquini C., Alfonsi F., Marchio V., Gallo F., Zingari F., Bolzoni A.R., Romeggio S., Barone A. (2023). The Use of Zirconia for Implant-Supported Fixed Complete Dental Prostheses: A Narrative Review. Dent. J..

[6] Mackert J., El-Shewy M., Pannu D., Schoenbaum T. (2024). Prosthetic complications and survival rates of metal-acrylic implant fixed complete dental prostheses: A retrospective study up to 10 years. J. Prosthet. Dent..

[7] Estrin N., Nam K., Romanos G.E., Saragossi J., Iacono V.J., Bassir S.H. (2023). Clinical Outcomes of Metal-Ceramic versus Metal-Acrylic Resin Implant-Supported Fixed Complete Dental Prostheses: A Systematic Review and Meta-analysis. Int. J. Prosthodont..

[8] Abad-Coronel C., Vélez Chimbo D., Lupú B., Pacurucu M., Fárez M.V., Fajardo J.I. (2023). Comparative Analysis of the Structural Weights of Fixed Prostheses of Zirconium Dioxide, Metal Ceramic, PMMA and 3DPP Printing Resin—Mechanical Implications. Dent. J..

[9] Rammelsberg P., Meyer A., Lorenzo-Bermejo J., Kappel S., Zenthöfer A. (2021). Long-term chipping and failure rates of implant-supported and combined tooth–implant-supported metal-ceramic and ceramic fixed dental prostheses: A cohort study. J. Prosthet. Dent..

[10] Barootchi S., Askar H., Ravidà A., Gargallo-Albiol J., Travan S., Wang H.L. (2020). Long-term Clinical Outcomes and Cost-Effectiveness of Full-Arch Implant-Supported Zirconia-Based and Metal-Acrylic Fixed Dental Prostheses: A Retrospective Analysis. Int. J. Oral Maxillofac. Implants.

[11] Kocaağaoğlu H., Albayrak H., Kilinc H.I., Gümüs H. (2017). Effect of repeated ceramic firings on the marginal and internal adaptation of metal-ceramic restorations fabricated with different CAD-CAM technologies. J. Prosthet. Dent..

[12] Usta Kutlu İ., Hayran Y. (2024). Influence of various fabrication techniques and porcelain firing on the accuracy of metal-ceramic crowns. BMC Oral Health.

[13] Barbin T., Silva L.D.R., Velôso D.V., Borges G.A., Presotto A.G.C., Barão V.A.R., Groppo F.C., Ferraz Mesquita M. (2020). Biomechanical behavior of CAD/CAM cobalt-chromium and zirconia full-arch fixed prostheses. J. Adv. Prosthodont..

[14] Dib Zakkour S., Dib Zakkour J., Guadilla Y., Montero J., Dib A. (2023). Comparative Evaluation of the Digital Workflow and Conventional Method in Manufacturing Complete Removal Prostheses. Materials.

[15] Mobarak M.H., Islam M.A., Hossain N., Al Mahmud M.Z., Rayhan M.T., Nishi N.J., Chowdhury M.A. (2023). Recent advances of additive manufacturing in implant fabrication—A review. Appl. Surf. Sci. Adv..

[16] Drago C., Howell K. (2012). Concepts for designing and fabricating metal implant frameworks for hybrid implant prostheses. J. Prosthodont..

[17] Larsson C., Wennerberg A. (2014). The clinical success of zirconia-based crowns: A systematic review. Int. J. Prosthodont..

[18] Xiang Z.-X., Chen X.-P., Song X.-F., Yin L. (2020). Responses of pre-crystallized and crystallized zirconia-containing lithium silicate glass ceramics to diamond machining. Ceramics Int..

[19] Matta R.E., Berger L., Loehlein M., Leven L., Taxis J., Wichmann M., Motel C. (2024). Stress Distribution within the Peri-Implant Bone for Different Implant Materials Obtained by Digital Image Correlation. Materials.

[20] Lopez C.A.V., Vasco M.A.A., Ruales E., Bedoya K.A., Benfatti C.M., Bezzon O.L., Deliberador T.M. (2018). Three-Dimensional Finite Element Analysis of Stress Distribution in Zirconia and Titanium Dental Implants. J. Oral Implantol..

[21] Escobar M., Henriques B., Fredel M.C., Silva F.S., Özcan M., Souza J.C.M. (2020). Adhesion of PEEK to resin-matrix composites used in dentistry: A short review on surface modification and bond strength. J. Adhes. Sci. Technol..

[22] Gouveia D.d.N.M., Razzoog M.E., Sierraalta M., Alfaro M.F. (2021). Effect of surface treatment and manufacturing process on the shear bond strength of veneering composite resin to polyetherketoneketone (PEKK) and polyetheretherketone (PEEK). J. Prosthet. Dent..

[23] Sacks G., Shah V., Yao L., Yan C., Shah D., Limeta L., DeStefano V. (2024). Polyaryletherketones: Properties and applications in modern medicine. Biomed. Technol..

[24] Ahmad F., Nimonkar S., Belkhode V., Nimonkar P. (2024). Role of Polyetheretherketone in Prosthodontics: A Literature Review. Cureus.

[25] Schwitalla A., Muller W.D. (2013). PEEK dental implants: A review of the literature. J. Oral Implantol..

[26] Schwitalla A.D., Spintig T., Kallage I., Muller W.D. (2016). Pressure behavior of different PEEK materials for dental implants. J. Mech. Behav. Biomed. Mater..

[27] Bathala L., Majeti V., Rachuri N., Singh N., Gedela S. (2019). The Role of Polyether Ether Ketone (Peek) in Dentistry—A Review. J. Med. Life.

[28] Reda R., Zanza A., Galli M., De Biase A., Testarelli L., Di Nardo D. (2022). Applications and Clinical Behavior of BioHPP in Prosthetic Dentistry: A Short Review. J. Compos. Sci..

[29] Luo C., Liu Y., Peng B., Chen M., Liu Z., Li Z., Kuang H., Gong B., Li Z., Sun H. (2023). PEEK for Oral Applications: Recent Advances in Mechanical and Adhesive Properties. Polymers.

[30] Alsadon O., Moorehead R., Almansour H., Bangalore D., Alageel O., Wood D. (2023). Surface Characteristics and Adhesion of Veneering Composite Resin to PAEK-Based Substructure Restorative Materials. J. Prosthodont..

[31] Wang B., Huang M., Dang P., Xie J., Zhang X., Yan X. (2022). PEEK in Fixed Dental Prostheses: Application and Adhesion Improvement. Polymers.

[32] Jin H.Y., Teng M.H., Wang Z.J., Li X., Liang J.Y., Wang W.X., Jiang S., Zhao B.D. (2019). Comparative evaluation of BioHPP and titanium as a framework veneered with composite resin for implant-supported fixed dental prostheses. J. Prosthet. Dent..

[33] Jovanović M., Živić M., Milosavljević M. (2021). A potential application of materials based on a polymer and CAD/CAM composite resins in prosthetic dentistry. J. Prosthodont. Res..

[34] Bechir E., Bechir A., Cherana G., Manu R., Burcea A., Dascalu I. (2016). The Advantages of BioHPP Polymer as Superstructure Material in Oral Implantology. Mater. Plast..

[35] Alzahrani S.J., Hajjaj M.S., Azhari A.A., Ahmed W.M., Yeslam H.E., Carvalho R.M. (2023). Mechanical Properties of Three-Dimensional Printed Provisional Resin Materials for Crown and Fixed Dental Prosthesis: A Systematic Review. Bioengineering.

[36] Cevik P., Schimmel M., Yilmaz B. (2022). New generation CAD-CAM materials for implant-supported definitive frameworks fabricated by using subtractive technologies. Biomed. Res. Int..

[37] Yeslam H.E. (2023). Flexural Behavior of Biocompatible High-Performance Polymer Composites for CAD/CAM Dentistry. J. Compo Sci..

[38] Soares P.M., Cadore-Rodrigues A.C., Packaeser M.G., Bacchi A., Valandro L.F., Pereira G.K.R., Rippe M.P. (2022). Masking ability of implant abutment substrates by using different ceramic restorative systems. J. Prosthet. Dent..

[39] Youssef M.K., Abdelkader S.H., Aly Y.M. (2023). Effect of different interfacial surface treatments on the shear bond strength of veneering ceramic and zirconia core. BMC Oral Health.

[40] Kiliç M., Dede D.Ö., Küçükekenci A.S. (2023). Comparing the shear bond strength of veneering materials to the PAEKs after surface treatments. BMC Oral Health.

[41] Sarfaraz H., Rasheed M.N., Shetty S.K., Prabhu U.M., Fernandes K., Mohandas S. (2020). Comparison of the Bond Strength of Composite Resin to Zirconia and Composite Resin to Polyether Ether Ketone: An In Vitro Study. J. Pharm. Bioallied Sci..

[42] Sloan R., Hollis W., Selecman A., Jain V., Versluis A. (2022). Bond strength of lithium disilicate to polyetheretherketone. J. Prosthet. Dent..

[43] Bioloren Trilor^®^ Disks-Discs in Hi-Tech Material for Dental Prosthesis Without Metal. https://bioloren.com/english/trilor-fiber-disks-and-blocks/trilor-disks#.

[44] Bredent BioHPP^®^-A Biocompatible, Strong High Performance Polymer for Dental Prosthetics. https://www.bredent.co.uk/products/bionic-framework-materials/benefits-of-biohpp/.

[45] Gökay G.D., Aladağ S.Ü. (2024). Comparison of the shear bond strengths of two different polyetheretherketone (PEEK) framework materials and CAD–CAM veneer materials. BMC Oral Health.

[46] Alsarani M.M., Bangalore D., Alfrisany N., Alshamrani A., El-Bialy T. (2024). Effect of conventional and digital methods and aging on the shear bond strength of orthodontic brackets with temporary crowns based on aged PMMA. Polimery.

[47] (2020). Dentistry—Polymer-Based Crown and Veneering Materials.

[48] Morresi A.L., D’Amario M., Capogreco M., Gatto R., Marzo G., D’Arcangelo C., Monaco A. (2014). Thermal cycling for restorative materials: Does a standardized protocol exist in laboratory testing? A literature review. J. Mech. Behav. Biomed. Mater..

[49] Adeeb Gabra E.N., Sadek H.M.A., Hamdy A.M., Wahsh M.M. (2024). Effect of surface treatment and resin cement type on the bond strength of polyetheretherketone to lithium disilicate ceramic. BMC Oral Health.

[50] Khaohoen A., Sornsuwan T., Chaijareenont P., Poovarodom P., Rungsiyakull C., Rungsiyakull P. (2023). Clinical Medicine Review Biomaterials and Clinical Application of Dental Implants in Relation to Bone Density—A Narrative Review. J. Clin. Med..

[51] Seong W.J., Kim U.K., Swift J.Q., Heo Y.C., Hodges J.S., Ko C.C. (2009). Elastic properties and apparent density of human edentulous maxilla and mandible. Int. J. Oral Maxillofac. Surg..

[52] Bechir F., Bataga S.M., Tohati A., Ungureanu E., Cotrut C.M., Bechir E.S., Suciu M., Vranceanu D.M. (2021). Evaluation of the Behavior of Two CAD/CAM Fiber-Reinforced Composite Dental Materials by Immersion Tests. Materials.

[53] Çulhaoğlu A.K., Özkır S.E., Şahin V., Yılmaz B., Kılıçarslan M.A. (2020). Effect of Various Treatment Modalities on Surface Characteristics and Shear Bond Strengths of Polyetheretherketone-Based Core Materials. J. Prosthodont..

[54] Erjavec A.K., Črešnar K.P., Švab I., Vuherer T., Žigon M., Brunčko M. (2023). Determination of Shear Bond Strength between PEEK Composites and Veneering Composites for the Production of Dental Restorations. Materials.

[55] Gouda A., Sherif A., Wahba M., Morsi T. (2023). Effect of veneering material type and thickness ratio on flexural strength of bi-layered PEEK restorations before and after thermal cycling. Clin. Oral Investig..

[56] Ates S.M., Caglar I., Yesil Duymus Z. (2018). The effect of different surface pretreatments on the bond strength of veneering resin to polyetheretherketone. J. Adhes. Sci. Technol..

[57] Bunz O., Benz C.I., Arnold W.H., Piwowarczyk A. (2021). Shear bond strength of veneering composite to high performance polymers. Dent. Mater. J..

